# Vitamin A – discovery, metabolism, receptor signaling and effects on bone mass and fracture susceptibility

**DOI:** 10.3389/fendo.2024.1298851

**Published:** 2024-04-22

**Authors:** Ulf H. Lerner

**Affiliations:** Department of Internal Medicine and Clinical Nutrition, Institute of Medicine, Sahlgrenska Osteoporosis Centre and Centre for Bone and Arthritis Research at the Sahlgrenska Academy, University of Gothenburg, Gothenburg, Sweden

**Keywords:** vitamin A, bone, osteoporosis, osteoclast, osteoblast

## Abstract

The first evidence of the existence of vitamin A was the observation 1881 that a substance present in small amounts in milk was necessary for normal development and life. It was not until more than 100 years later that it was understood that vitamin A acts as a hormone through nuclear receptors. Unlike classical hormones, vitamin A cannot be synthesized by the body but needs to be supplied by the food as retinyl esters in animal products and ß-carotene in vegetables and fruits. Globally, vitamin A deficiency is a huge health problem, but in the industrialized world excess of vitamin A has been suggested to be a risk factor for secondary osteoporosis and enhanced susceptibility to fractures. Preclinical studies unequivocally have shown that increased amounts of vitamin A cause decreased cortical bone mass and weaker bones due to enhanced periosteal bone resorption. Initial clinical studies demonstrated a negative association between intake of vitamin A, as well as serum levels of vitamin A, and bone mass and fracture susceptibility. In some studies, these observations have been confirmed, but in other studies no such associations have been observed. One meta-analysis found that both low and high serum levels of vitamin A were associated with increased relative risk of hip fractures. Another meta-analysis also found that low levels of serum vitamin A increased the risk for hip fracture but could not find any association with high serum levels of vitamin A and hip fracture. It is apparent that more clinical studies, including large numbers of incident fractures, are needed to determine which levels of vitamin A that are harmful or beneficial for bone mass and fracture. It is the aim of the present review to describe how vitamin A was discovered and how vitamin A is absorbed, metabolized and is acting as a ligand for nuclear receptors. The effects by vitamin A in preclinical studies are summarized and the clinical investigations studying the effect by vitamin A on bone mass and fracture susceptibility are discussed in detail.

## Introduction

Vitamin A, present in both animal products and vegetables, is a fat-soluble compound referring to substances possessing the biological activity of retinol and characterized by unsaturated isoprenoid chain structures. It is well known that vitamin A is important for normal growth, reproduction, vision and immunity, but also for having important roles in pathological processes such as cardiovascular diseases, cancer, skin diseases and obesity (1). High levels of vitamin A have also been considered as a risk factor for secondary osteoporosis (2, 3). Biological effects by vitamin A are due to activation of nuclear receptors by the retinol metabolite all-*trans*-retinoic acid (ATRA) and subsequent regulation of more than 500 genes in a variety of cell types (4). Although the main aim of the present review is to discuss skeletal remodeling as a consequence of high levels of vitamin A in industrialized countries, it is important to note that nutritional deficiency of vitamin A is a huge public health problem worldwide. In developing countries, in which the prevalence of vitamin A deficiency can be as common as 60% of the population, the deficiency causes xerophthalmia and blindness in more than 4 million children and numerous numbers of early deaths due to infections (5).

### Discovery

As pointed out by Semba (6), it is not possible to establish when vitamin A was discovered and who made the discovery. Important early observations were made by the Russian scientist Nicolai Lunin 1881 in experiments where adult mice were found to live well on milk, but were unable to stay alive on a diet containing protein, fat, carbohydrates, salt and water unless this food was supplemented with dried milk (7). Shortly afterwards (1891), it was found that unknown, fat-like substance(s) in egg yolk was/were necessary for good health in mice (8). Further support for the finding that milk contained important substance(s) was the observation 1909 that rats fed a diet containing the essential nutrients for life according to the prevailing dogma, *i.e.* protein, fat, carbohydrates and minerals, died, but stayed alive healthy if milk was added to this diet (9). The most famous experiments were then made by the British physician and biochemist Frederic Gowland Hopkins (1912), who reported that young rats given protein, starch, cane sugar, lard and minerals gained weight considerably less than rats fed the same diet with very small amounts of milk (10). He also showed that when milk was withdrawn from the food given to well growing rats, these rats stopped growing, and that the slowly growing rats started to grow fast when they were given food with milk. He concluded that an “accessory factor” present in “astongishly small amounts” in milk is essential for life, although these studies did not show which substance or substances that were important. The same year, Casmir Funk suggested to designate all minor essential nutrients in foods as ´vitamins´, with the mistaken idea that all these factors are ´vital amines´ (11), which led to that the “accessory food factor”, sometimes called “fat-soluble A”, eventually was given the name “vitamin A”.

Sir Frederic Gowland Hopkins shared the Noble Prize in Medicine and Physiology 1929 for having discovered vitamins with the Dutch physician Christiaan Eijkman, who had described that beriberi could be cured by a compound present in rice (now known as thiamin or vitamin B1). Hopkins was, however, always keen to give credit to the early observations by Nicolai Lunin (7).

By feeding vitamin A deficient mice with carotenoids, it was shown that carotene is a precursor to vitamin A (12). The Swiss chemist Paul Karrer described the chemical formula of carotenoid and later showed how carotenoids can be transformed to vitamin A, observations followed by the establishment of the chemical structure of vitamin A itself using extracts from cod-liver oil (13, 14). Paul Karrer received the Noble Prize in Chemistry 1937 for these achievements. Vitamin A was crystallized 1937 (15) and during late 1940´s vitamin A was synthesized and produced in large scale to be used for treatment of deficiency (16, 17).

During the following two decades, several groups reported that deficiency and excess of vitamin A resulted in developmental disturbances. An important finding during this time period was the demonstration that 11-*cis*-retinal, together with the protein opsin, makes up the light sensitive chromophore rhodopsin in the eye (18), a finding which resulted in that the American biochemist George Wald was awarded the Noble Prize in Physiology and Medicine 1967 together with the Finnish physiologist Ragnar Granit and the American physiologist Haldan Keffer Hartline. A key event in vitamin A biology was the discoveries by the American biochemist Hector deLuca that all-*trans*-retinoic acid (ATRA) is formed *in vivo* from retinol and in fact is a natural metabolite of retinol (19, 20), followed by the observation that ATRA is the most potent metabolite of vitamin A inducing differentiation of F9 embryonal carcinoma cells *in vitro* (21). During the 1970´s, binding proteins for retinol and retinoic acid in plasma and cells were discovered, followed by studies showing how retinoids were metabolized and transported extra- and intracellularly (22, 23). Subsequent studies showed that some intracellular binding proteins function as nuclear receptors, which when binding to DNA act as transcription factors following dimerizing with other nuclear receptors (24–26).

### Beneficial effects by vitamin A

A large number of different cell types express receptors for vitamin A and thereby are target cells for this pleiotropic hormone. In many of these cells, vitamin A regulates cell growth and differentiation and is important both for embryological development, including of the skeleton (see further below “Vitamin A receptor signaling”), as well as for tissue growth and regeneration during postnatal life (27, 28). Importantly for these functions, vitamin A regulates subsets of genes in stem cells involved in their fate and differentiation (29).

In hematopoiesis, vitamin A enhances expansion of hematopoietic stem cells and induces differentiation of granulocytes from committed myeloid precursors (30). Vitamin A is important in immune processes in both innate and acquired immunity through effects on regulatory T cell growth and differentiation, B-cell maturation and antibody responses and differentiation of dendritic cells (31–33).

Vitamin A has also important effects in a variety of different functions in epithelial cells in tissues such as skin, sweat glands and salivary glands. Effects on keratinocytes involves stimulation of epithelial cells growth and differentiation during wound healing (33).

Due to effects by vitamin A on cell proliferation, differentiation and apoptosis, a lot of interest has been directed to a potential role of vitamin A in cancer and both stimulatory and inhibitory effects have been observed. However, treatment of certain cancers with different retinoids, including ATRA, is approved by FDA (33). Treatment of acute promyelocytic leukemia (APL) has turned out to be particularly successful (34). Retinoids are also used to treat skin diseases such as psoriasis, acne, seborrhea and ichthyosis (33).

### Harmful effects by vitamin A

Acute harmful effects by excess of vitamin A are rare today but are well known among early Arctic explorer who suffered from vertigo, vomiting, diarrhea, headache and convulsions which could cause their death when consuming vitamin A rich liver from polar beer or from Greenland Husky sled dog. Today, hypervitaminosis A is sometimes observed in young people consuming large amounts of candy-like supplements or overdosing retinoids for treatment of acne (35).

Deleterious effects by vitamin A on the skeleton observed in preclinical studies was reported already 1934 (36). Extreme softness of bone and spontaneous fractures were observed in rats given high doses of vitamin A. During the 1940´s, limping gait and fractures associated with increased bone resorption were demonstrated in rats and guinea pigs given high amounts of vitamin A (37, 38). Similar observations were made in dogs given vitamin A (39). It was not known if the deleterious effects by vitamin A on the skeleton were caused by direct or indirect effects. Barnicot was the first to show 1948 a direct erosive effect on bone by adding fragments of crystalline vitamin A on parietal bones *ex vivo*, which were then transplanted to cerebral hemispheres of littermates (40). Direct effect by vitamin A on bone loss associated with osteoclasts was also noted in explant cultures of either chicken limb bone or fetal mouse long bones (41, 42). Since then, numerous studies have demonstrated increased bone resorption, decreased bone mass and enhanced fracture susceptibility in rodents given high doses of vitamin A for short period of time (2).

The first clinical reports describing effects by vitamin A on the skeleton in humans were some few case reports published during 1944 -1988 describing symptoms in patients taking intoxicating doses of vitamin A (43). These reports described bone pain, radiographic osteopenia, hypercalcemia and increased bone resorption. Early epidemiological studies during 1980 and 90’s yielded contradictory results when assessing possible associations between dietary intake of vitamin A, serum retinol and bone mass (43). These studies, however, were often low-powered with less accurate methods to assess bone mass. The first large, well designed clinical study was the investigation by Melhus et al. in which dietary intake of retinol was found to be positively related to decreased bone mass in several skeletal sites, using dual-energy X-ray absorptiometry (DXA), and to increased hip fracture risk (44). Since then, several studies assessing the potentially detrimental effect by vitamin A on bone mass and fracture risk have been published, however, with divergent results (2).

### Vitamin A metabolism and receptor signaling

Since vitamin A is not produced by the body it needs to be provided in the diet either as retinyl esters in animal products or as ß-carotene in vegetables. These precursor molecules are taken up by the intestine and then delivered to the lymphatics before being released into blood. A substantial part of vitamin A is stored in the liver and serves as a reservoir in case of vitamin A deficiency. In the circulation, vitamin A is present mainly as retinol but also as retinyl esters and ß-carotene. Uptake and metabolism of vitamin A involve several binding proteins and enzymes.

To regulate target cell activities, circulating forms of vitamin A need to be converted in the cytoplasm to the biologically active compound ATRA, which acts as a ligand for vitamin A receptors in the nucleus. These receptors heterodimerize with another type of nuclear vitamin A receptor for which the ligand is elusive. The complex binds to DNA and either activate or repress gene transcription.

It is the aim of the present review to briefly describe the metabolism and cellular effects by vitamin A and to present pro and cons regarding the potential harmful effect by vitamin A on the skeleton and to argue for the need of well-powered prospective clinical studies including large numbers of incident fractures. It is also suggested that the potential effects by vitamin A on load-induced anabolic response, fracture healing and effects by bone anabolic drugs should be investigated.

## Vitamin A metabolism

Since vitamin A cannot be synthesized in the body, precursor molecules need to be acquired from the food and converted to biologically active compounds through many steps in the intestine, liver and, finally, in target cells. These processes involve several enzymes, binding proteins as well as cell surface and nuclear receptors. In the food, vitamin A is present as retinyl esters (mainly retinyl palmitate) in animal products such as egg, meat, fish, fortified cereals and dairy products, and in plant-based food such as vegetables and fruits as ß-carotene.

**Figure 1:**
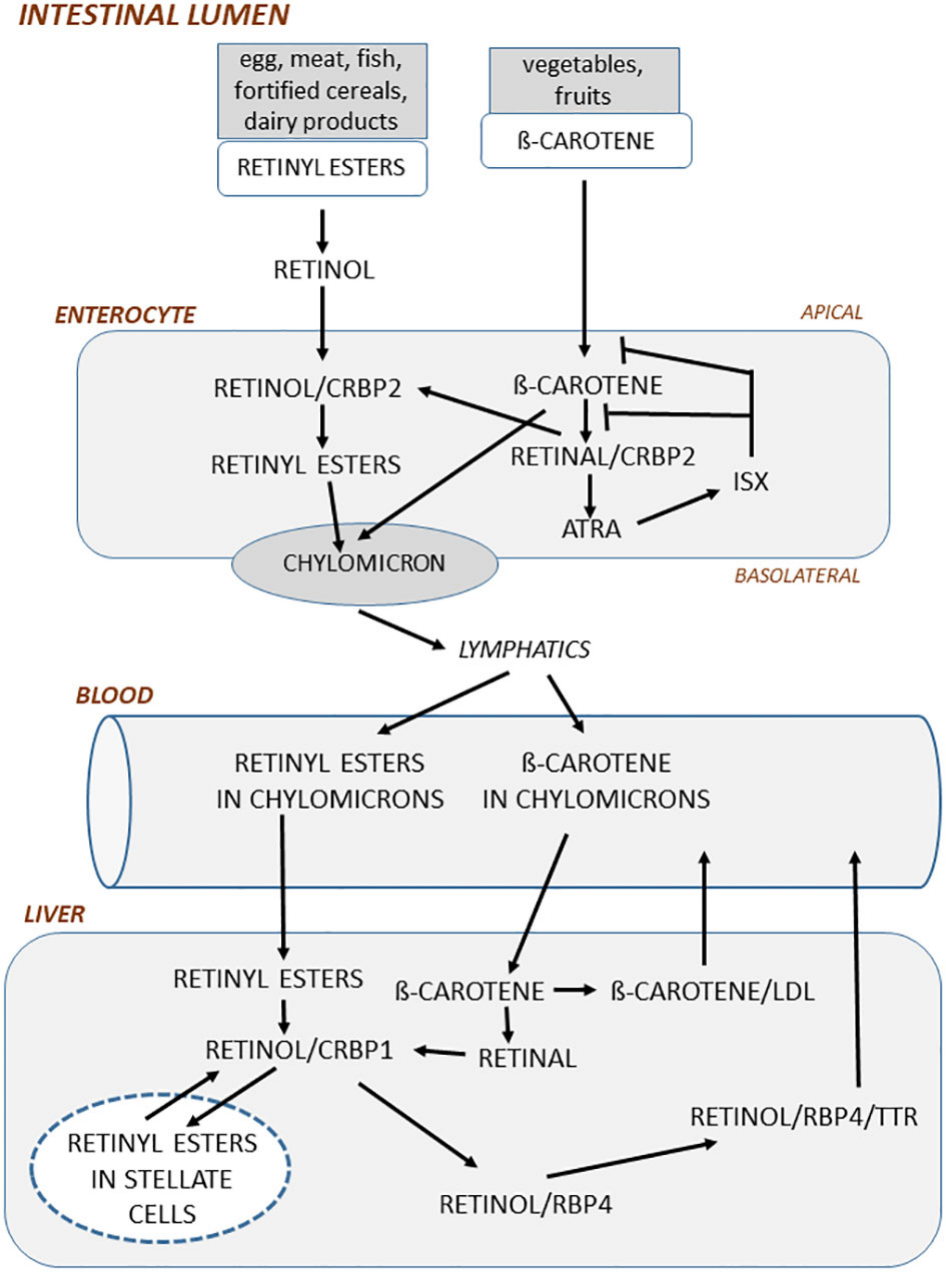
In the intestinal lumen, retinyl esters in food is hydrolyzed to retinol mainly by pancreatic enzymes. Retinol is taken up by enterocytes in the apical side of enterocytes and then bound to cellular retinol-binding protein 2 (CRBP2) and subsequently esterified by lechitin retinol acyltransferase to retinyl esters. ß-carotene in the food is taken up by enterocytes and then oxidized to retinal by ß-carotene-15,15´-monooxygenase. Retinal bound to CRBP2 is then either converted to retinol by retinal reductases or oxidized to ATRA by retinal aldehyde dehydrogenase. ATRA induces the transcription factor ISX, which acts as a negative feedback regulator by inhibiting both conversion of ß-carotene to retinal and the uptake of ß-carotene. In the basolateral side of enterocytes, retinyl esters and non-converted ß-carotene are incorporated into chylomicrons, which through the lymphatics are delivered to blood. Retinyl esters in chylomicrons are taken up by the liver and there hydrolyzed to retinol by retinyl ester hydrolase. Retinol bound to CRBP1 is then either converted to retinyl esters by lechitin retinol acyltransferase and stored in lipid droplets in stellate cells or transported to the site where retinol is bound to retinol-binding protein 4 (RBP4). Before being delivered to blood, retinol/RBP4 is bound to transthyretin. ß-carotene taken up by liver is converted to retinal by ß-carotene oxygenase 1 and then further to retinol or bound to very low-density lipoprotein and released to blood.

Since retinyl esters and ß-carotene are water insoluble they are transferred in the intestinal lumen to mixed micelles, a process which is facilitated by gastric and pancreatic enzymes. Before being taken up by enterocytes, retinyl esters are hydrolyzed to retinol mainly by pancreatic lipase. The uptake of retinol and ß-carotene in the apical side of enterocytes is not known in detail but is believed to be due to both passive diffusion and facilitated transport (45) (**Figure 1**). Importantly, the uptake and intracellular metabolism of ß-carotene in enterocytes are under regulation of a negative feed-back loop controlled by the transcription factor ISX (intestinal-specific homeobox gene), which is induced by ATRA (46).

In the enterocytes, retinol binds to CRBP2 (cellular retinol-binding protein 2), a binding protein solely expressed in intestine (47), and is then esterified to retinyl esters, which is necessary for the incorporation in chylomicrons (small particles of triglycerides, phospholipids, proteins, and cholesterol), whereas ß -carotene is first converted to retinal, then to retinol and subsequently to retinyl esters (48) (**Figure 1**). The retinyl esters and some non-converted ß-carotene are packed in chylomicrons, which are delivered through the basolateral part of enterocytes to the lymphatics and then released into blood. ATRA is also formed in the enterocytes which induces ISX through nuclear receptors (see further below).

A unique feature of vitamin A is that, unlike other vitamins, vitamin A can be stored in the body. Although several organs and cells can store vitamin A, including fat cells, the main part (70%) of vitamin A is stored in the liver (23). Storage of vitamin A in the body can protect from vitamin A insufficiencies for several months. Following uptake of chylomicrons in the liver, retinyl esters and ß-carotene are released into hepatocytes, where retinyl esters are hydrolyzed to retinol and bound to CRBP1 (49) (**Figure 1**). Retinol is then either bound to RBP4 (retinol-binding protein 4) or transported to fat-storing hepatic stellate cells and esterified to retinyl esters (50, 51). 70-90% of vitamin stored in the liver is as retinyl esters in stellate cells. How ß-carotene is metabolized in the liver is less known, but it is assumed that it is converted to retinal and then to retinol and retinyl esters and subsequently either stored in hepatic cells or bound to RBP4 as retinol. Secretion of vitamin A from the liver is mainly as retinol/RBP4, which is also bound to the transporter protein TTR (transthyretin) to stabilize the complex and to reduce filtration through the kidneys (52).

In the circulation, a variety of different forms of vitamin A are present, including retinol, retinyl esters, carotenoids and retinoic acid, which concentrations can vary at fasting state and during postprandial periods (23). The concentration of retinol/RBP4/TTR is tightly regulated and varies between 2 and 4 µM, with the exception of in vitamin A deficient individuals in whom the concentration can be significantly increased after a vitamin A reach meal (23, 53–55). Serum levels of retinol <0.7 µM indicate vitamin A deficiency, whereas levels >2.1 µM are suggested to indicate hypervitaminosis A (33). Similar to retinol, ß-carotene in blood is regulated and the concentration is 5-8 µM (56). The concentration of retinyl esters in blood, however, varies considerably from 100-200 nM during fasting conditions up to 5-10 µM at postprandial periods (57). The ratio of fasting serum retinyl esters to total serum vitamin A (retinol+retinyl esters) has also been suggested to be a marker of vitamin A status. A ratio >10% has been suggested as a marker of potential vitamin A toxicity (58, 59). Retinoic acid is present in blood at 1-3 nM, but can increase for a short period of time to 80-90 nM after a vitamin A rich meal (60). As discussed in more detail by Borel and Desmarchelier (2017), the concentrations of different retinoids in blood are modulated by a large number of proteins, which participate in the metabolism of chylomicrons and other lipoproteins transporting ß-carotene and retinyl esters and by those proteins which are important for liver secretion and blood metabolism of retinol (48). Moreover, the bioavailability of vitamin A is affected more by vitamin A stores than by serum retinol levels (61) and is also affected by genetic variability as assessed by genome-wide association studies (48).

**Figure 2:**
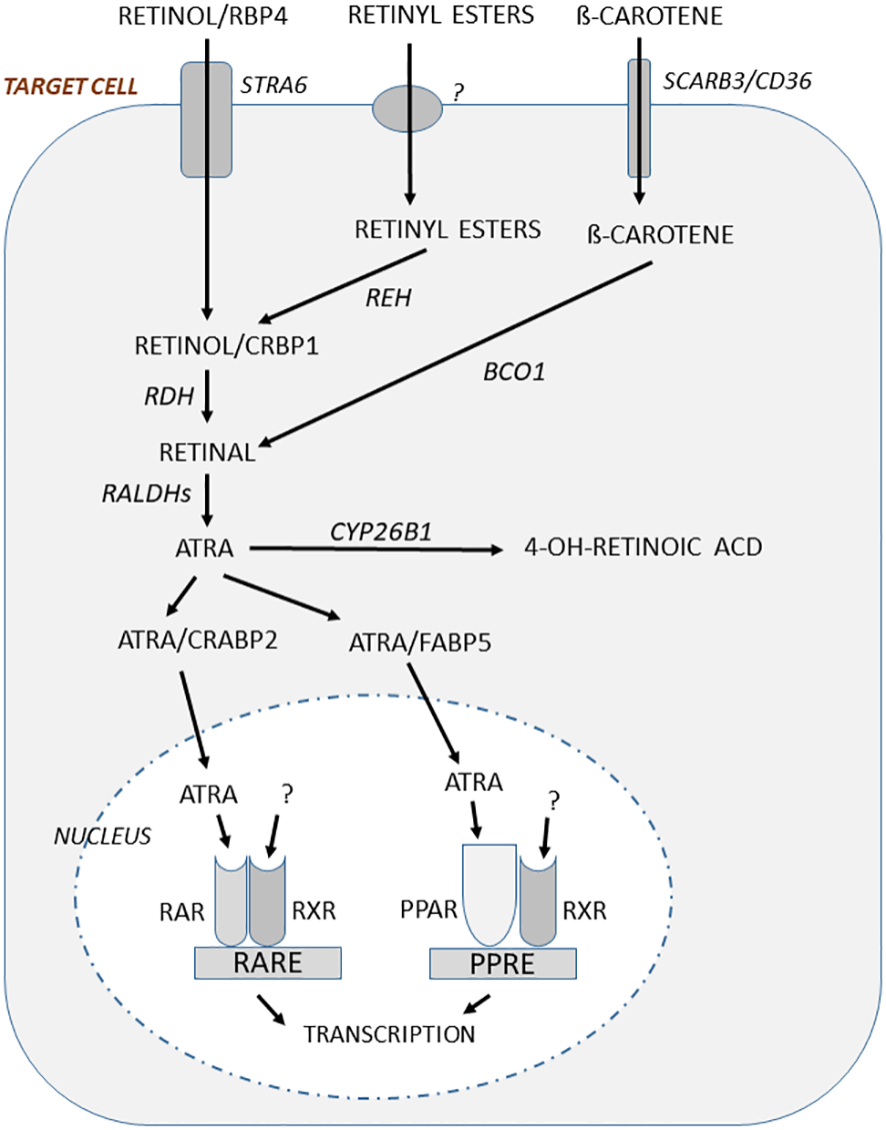
In target cells responding to regulation by vitamin A, retinol bound to RBP4 is taken up through the receptor STRA6 and then bound to CRBP1, whereas ß-carotene uptake is mediated by SCARB3/CD36. Retinyl esters are also taken up, but the mechanism is not known. The retinyl esters are hydrolyzed to retinol by retinyl ester hydrolase (REH). Retinol is oxidized by retinol dehydrogenase (RDH) to retinal which then is oxidized in a two-step processes using retinal aldehyde dehydrogenases (RALDH1, RALDH2) to form ATRA. Retinal is also formed by conversion of ß-carotene through ß-carotene-15,15´-monooxygenase (BCO1). Binding of ATRA to cellular retinoid acid-binding protein 2 (CRABP2) facilitates translocation to nucleus where ATRA binds to RARs. This complex heterodimerize with RXRs for which the ligand is not known, but may be 9-*cis*-13,14-dihydroretinoic acid. This dimeric ligand-inducible transcription factor will increase or decrease gene transcription through retinoic acid responsive elements (RAREs). ATRA can also bind to fatty acid-binding protein 5 (FABP5) and this complex facilitates translocation to the nucleus and binding to peroxisome proliferator-activated receptors (PPAR), which after heterodimerizing with RXRs act as a transcription factor increasing or decreasing gene transcription through peroxisome proliferator response element (PPRE).

In target cells regulated by vitamin A, there is some evidence that retinol/RBP4 is taken up by the cells through diffusion (62–64), but there is also evidence indicating that uptake can be facilitated through a receptor named STRA6 (stimulated by retinoic acid 6) (65, 66) (**Figure 2**). This multimembrane domain protein binds RBP4 and increases cellular uptake of retinol (65). The expression of STRA6 is crucial for the formation of rhodopsin in the eye, as shown by the decreased visual response in *Stra6*-deficient mice (67), similar to mice lacking *Rbp4* (68). STRA6, however, seems not to be important for uptake of retinol in all cell types, since although *Stra6* is expressed in several cell types, there are several cells which lack *Stra6* expression. Human bone marrow stromal cells have been shown to express *STRA6* mRNA and the expression seemed to be higher in cells isolated from osteoporotic patients compared to cells from osteopenic or healthy individuals (69). The authors also reported that *Stra6* mRNA was expressed in the mouse embryonic fibroblast-like cell line C3H10T1/2 and the mouse calvarial osteoblast cell line MC3T3/E1. Some evidence was presented indicating that *Stra6* could regulate osteoblast differentiation, but the results are difficult to interpret since no information is given whether retinol/RBP4 was present in the culture media. Therefore, the role of STRA6 for retinoid-dependent effects on bone cells is not known.

The uptake in target cells of ß-carotene present in blood in chylomicrons or bound to other lipoproteins is mediated by SCARB3 (scavenger receptors class B), also known as CD36 and the related SCARB1. It is not known if retinyl esters in chylomicron or bound to other lipoproteins are taken up through a receptor. It has, however, been suggested that retinyl esters can be taken up in target cells by a “flip-flop” process across the phospholipid bilayer in cell membranes (70) (**Figure 2**).

In the target cells, retinol delivered by uptake or formed after conversion of retinyl esters through retinyl ester hydrolase (REH), is bound to CRBP1 and then oxidized to retinal by retinol dehydrogenase (RDH) (**Figure 2**). Retinal is formed also by conversion of ß-carotene through ß-carotene-15,15´-monooxygenase (BCO1). Retinal is then converted by two-step oxidation processes through retinal aldehyde dehydrogenases (RALDH1, RALDH2) to ATRA, which is the biologically active form of vitamin A. After binding to CRABP2 (cellular retinoid acid-binding protein 2) ATRA is shuttled to the nuclei where it binds to retinoid acid receptors (RARs). Global deletion of *Raldh1* in mice has been reported to result in increased cortical thickness and a slight enhanced trabecular bone mass, indicating a deleterious effect by vitamin A on the skeleton (71). ATRA can also be shuttled to the nuclei after being bound to FABP5 (fatty acid-binding protein 5) and then ligated to PPARß/γ (peroxisome proliferator-activated receptor), another nuclear receptor which similar to RARs (see further below) dimerizes with retinoid X receptors (RXRs) (**Figure 2**).

The intracellular concentrations of ATRA are not only dependent on biosynthesis, but also on degradation. Enzymes in the cytochrome P450 family such as the enzyme CYP26B1 (cytochrome P450 family 26 subfamily B member 1) catalyze the degradation of ATRA to inactive, hydroxylated metabolites (72, 73)(**Figure 2**). Global deletion of *Cyp26b1* in mice, or mutation of *Cyp26b1* in zebrafish, causes multiple skeletal and craniofacial deformities (74, 75). Interestingly, some case reports from patients with different missense mutations in the CYP26B1 gene have shown the importance of this enzyme for skeletal and joint morphogenesis with a large number of skeletal and craniofacial deformities described in humans (75, 76). Several mutations are lethal, but some individuals survive beyond infancy and in these individuals, also the presence of gracile long bones have been described (77). Thus, mutations rendering CYP26B1 less catalytically active causing accumulation of ATRA intracellularly cause skeletal phenotypes similar to hypervitaminosis A.

## Vitamin A receptor signaling

The discovery of vitamin A receptors followed studies investigating how other fat soluble hormones such as estrogen and glucocorticoids bind to intracellular proteins and DNA (78). Initially, a receptor which could bind retinoic acid (RAR) and with the capacity to function as a ligand-inducible transcription factor was described 1987 independently by Pierre Chambon and Ronald Evans (79, 80). Recently, the background for the discoveries have been described in two fascinating papers by each group (25, 26). Shortly afterwards, Pierre Chambon and Magnus Phfahl cloned a second RAR (81, 82). The initially described receptor was given the name RARα and the second RARß. Then, Chambon and colleagues cloned a third receptor designated RARγ (83). These three receptors are encoded by different genes in different chromosomes and due to alternative splicing several isoforms exist. Subsequent studies have shown the presence also of three related receptors, designated RXRα (84), RXRß (85) and RXRγ, which similar to RARs are encoded by different genes (86). The ligand binding domains in RARs and RXRs have only restricted homology, which explains why ATRA, the natural ligand for RARs, does not bind to RXR. 9-*cis*-retinoic acid is a high affinity ligand for both RARs and RXRs (87, 88), but since this compound is either undetectable or present in too low concentrations to activate RXR, 9-*cis*-retinoic acid is not likely to be an endogenous ligand for RXR. Recently, however, 9-*cis*-13,14-dihydroretinoic acid has been demonstrated to be an endogenous and physiologically relevant retinoid capable of activating RXRs (89). Interestingly, the two distinct retinoid receptor subfamilies were found to be closely related mechanistically since it was found that RXR was necessary for the DNA binding and transactivation by RAR. Not only was RXRs necessary for the activity of RARs but also for other nuclear receptors since RXRs act as co-factors for RARs, vitamin D receptor (VDR) and thyroid receptors (TR) by making up heterodimers with these receptors, a process necessary for DNA binding and transactivation of RARs, VDR and TR (90, 91). Later, it has been shown that RXRs make up heterodimers also with other nuclear receptors such as PPAR, Liver X receptor (LXR) and farnesoid X receptor (FXR) (92). This means that RXRs play a critical role for the activity of not only retinoids but also for certain other hormones since there will be a competition between ligands and receptors requiring RXRs as accessory factors.

The importance of the RARs for the physiological functions of vitamin A *in vivo* is supported by observations showing that mice deficient of *Rara* or *Rarb* (93, 94), as well as mice with *Rara*/*Rarg* double knockout (95) exhibit similar phenotypes as vitamin A deficient mice. The phenotypes observed in mice with deletion of *Rara/Rarg* is more severe and extensive compared to those found in vitamin A deficient mice, probably because of difficulties achieving absolute deprivation of vitamin A. In agreement with the well documented role for vitamin A in skeletal development, *Rara/Rarb* deficient mice have several abnormalities in craniofacial skeleton including lack of incisors, as well as malformations in vertebra and the appendicular skeleton (95). The phenotypes generated by deletion of *Rxrs*, however, have been difficult to interpret since RXRs heterodimerize with several other nuclear receptors such as RARs, PPARs, VDR, TR and LXR.

## Experimental studies on effects by vitamin A on bone resorption and bone mass

### Effects by vitamin A on bone resorption

**Figure 3:**
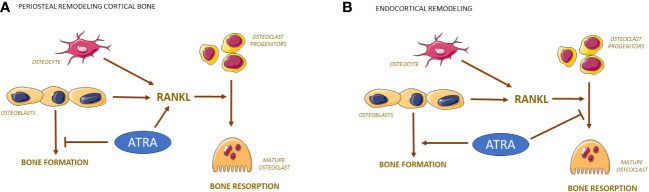
Bone resorption and formation are regulated differently by ATRA on periosteal and endocranial surfaces of bone. RANKL produced by osteocytes/osteoblasts activates the differentiation of mononuclear osteoclast progenitor cells to mature osteoclasts. On periosteal surfaces, ATRA induces the expression of RANKL causing enhanced number of osteoclasts while inhibiting the bone forming effects by osteoblasts **(A)**. On endocortical surfaces, ATRA inhibits the differentiation of osteoclast progenitors causing decreased number of osteoclasts while stimulating bone formation by osteoblasts **(B)**.

As mentioned in the introduction, there are several studies in rodents showing that large amounts of retinoids in the chow, or retinoids administered orally, or by *s.c*. injections in rats (96–101) or mice (99, 102) result in decreased cortical thickness, periosteal circumference and strength in the long bones. The loss of bone has been attributed to an increased formation of periosteal osteoclasts (**Figure 3A**), which together with findings of enhanced levels of hydroxyproline in urine and TRAP5b in serum show that the loss of bone is due to increased bone resorption. Mechanistically, enhanced bone resorption is caused by increased RANKL/OPG ratio induced by ATRA (103). Decreased cortical bone mass caused by increased amounts of vitamin A is also indicted by the observation that deletion in mice of *Raldh*, the intracellular enzyme responsible for the final step in formation of ATRA, results in increased cortical thickness, although it could not be established if this was due to decreased bone resorption or enhanced bone formation (71). Interestingly, increased numbers of osteoclasts have been observed in a patient with homozygous mutation of *CYB26B1*, the enzyme responsible for degradation of ATRA (104).

Studies aiming to elucidate if hypervitaminosis A also affects trabecular bone have yielded conflicting results. In rats, two studies have shown decreased trabecular bone mass in femur and tibia (100, 101), whereas three studies have not been able to observe any effect on trabecular bone mass in humerus or tibia (98, 99, 105). In mice fed with an increased vitamin A diet, Yorgan et al. observed reduced trabecular bone mass in vertebrae, associated with increased numbers of osteoclasts and decreased numbers of osteoblasts (106), whereas Lionikaite et al. could not observe any effect on trabecular bone mass in femur, tibia or vertebra in mice on increased vitamin A diet, although cortical bone mass was decreased (102, 107).

In only one study was investigated if hypervitaminosis A may affect also skull bones (108). The authors demonstrated that high dose of retinoids decreased the thickness of calvaria in rats, an effect due to both increased numbers of endocranial osteoclasts and decreased pericranial bone formation rate. Interestingly, also increased osteocyte lacuna was observed indicating that hypervitaminosis A may increase osteocytic osteolysis. In agreement with these experimental observations, thin skull bones has been described in young children suffering from vitamin A intoxication (109).

These studies unequivocally show that vitamin A strongly affects long bones causing decreased cortical bone mass, smaller bone with decreased diaphyseal diameter and decreased bone strength in rodents treated with high doses of retinoids for short period of time. It could, however, be questioned to what extent these results are of relevance for potential effects by vitamin A in humans with modest increases of vitamin A. With the aim to assess if also a lower dose of vitamin A would affect the skeleton, Johansson et al. fed rats with retinyl palmitate and retinyl acetate for seven days at a dose (1200 IU vitamin A/g pellet instead of 1700 IU/g pellet) which resulted in 18 times increased serum levels of retinyl ester from 270 to 5300 nM. It was observed that cortical bone area was still significantly decreased (98). In a more recent study, mice were fed with retinyl palmitate for 4 and 10 weeks with two doses of retinyl palmitate resulting in an increase of retinyl esters from 65 nM to 130 and 230 nM, respectively, after 10 weeks (102). By increasing serum retinyl esters 2 and 3.5 times, respectively, a dose- and time-dependent decrease of cortical thickness and periosteal circumference was observed, which was statistically significant at the highest dose. Linear regression analyses showed that cortical thickness in femur and periosteal circumference were significantly associated with the dosage of vitamin A. Since the effects were more pronounced after 10 than 4 weeks, the possibility exists that even larger effects could have been obtained if treatment time had been extended more than 10 weeks. Since the physiological levels of retinyl esters in humans are in the range of 50-200 nM (58, 59) these experiments indicate that clinically relevant doses of vitamin A have deleterious effects on the skeleton in mice.

### Effects by vitamin A on bone formation

Most of the experimental studies in rodents have focused on effects by vitamin A on bone resorption. There are, however, some studies also assessing effects on bone formation. The decreased periosteal circumference by increased levels of vitamin A seems to be due not only to increased periosteal bone resorption, but also to decreased bone formation (**Figure 3A**). Dynamic histomorphometry has demonstrated decreased periosteal bone formation rate in rats given high doses of vitamin A (110), and in mice treated with considerably smaller doses of vitamin A (102).

### Site specific effects by vitamin A on bone resorption and formation

Interestingly, vitamin A seems to have site specific effects on both bone formation and osteoclastogenesis. Decreased endocortical circumference and bone marrow area are observed in femur and tibia in both rats and mice with high and moderately increased doses of vitamin A (100, 102). This is to a large extent dependent on increased endocortical bone formation as assessed by both histology and dynamic histomorphometry (102, 104), but decreased osteoclast numbers on endocortical surfaces also contribute (**Figure 3B**) (100, 102). In contrast to the increase of endocortical bone formation, vitamin A decreases bone formation on periosteal surfaces on long bones and skull bones without affecting bone formation on endocranial and intracranial surfaces of the skull bones (102, 108, 110). The decrease of endocortical osteoclasts contrasts with the increased numbers of osteoclasts observed at periosteal surfaces in appendicular skeleton and endocranially in skull bones (99, 100, 102). Interestingly, vitamin A has no effect on osteoclasts numbers intracranially (108) in contrast to TNF-α, IL-1ß, PTH and 1,25(OH)_2_-vitamin D3, which decrease calvarial bone mass by increasing osteoclast numbers intracranially (111). Currently, it is not known the reason why vitamin A differently affects the response in osteoblasts and osteoclast in different parts of the skeleton.

### Effects by vitamin A on loading induced bone mass

**Figure 4:**
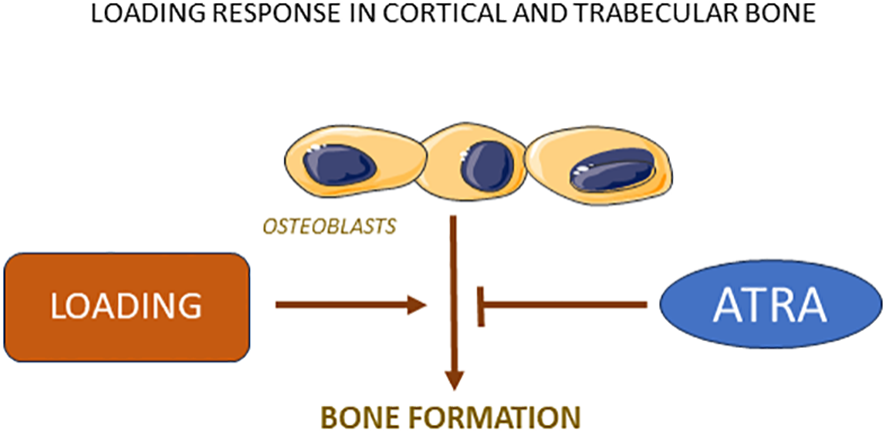
Loading stimulates bone formation in both cortical and trabecular bone due to increased osteoblast activity, a response which is robustly inhibited by ATRA.

In one study, the possibility has been assessed that vitamin A may affect the increase of bone mass caused by mechanical loading (107). For this purpose, axial loading of tibiae in mice was applied on alternate days for two weeks resulting in an 43% increase of trabecular bone mass. This response was substantially reduced by feeding the mice with retinoid palmitate at a dose causing a modest increase of retinyl esters. Loading increased also cortical thickness and pericortical circumference and both responses were decreased by increasing the dosage of vitamin A. The reduced anabolic response in mice with increased dosage of vitamin A was due to decreased bone formation rate (**Figure 4**). These findings may have implications for the gain of bone mass achieved by physical activity in humans. The observations should prompt studies investigating if vitamin A may affect bone formation during fracture healing or induced by anabolic drugs used for treatment of osteoporosis.

### Summary

In long bones, increasing amounts of vitamin A in rodents cause enhanced bone resorption and decreased bone formation on periosteal surfaces and decreased bone resorption and increased bone formation on endocortical surfaces, resulting in decreased cortical thickness, decreased diaphyseal diameter and enhanced fragility. In skull bones, bone density is decreased due to enhanced endocranial bone resorption, decreased precranial bone formation and enhanced osteocytic osteolysis. Increased bone mass caused by mechanical loading of long bones is reduced by vitamin A.

Although the relevance of experimental observations in rodents for human physiology and pathology should be discussed, the deleterious effects by increasing amounts of vitamin A for the skeleton in rats and mice, even at clinically relevant amounts, should be taken into account when evaluating possible associations between enhanced levels of vitamin A and osteoporosis in humans.

## Epidemiological studies investigating effects by vitamin A on the skeleton

Pioneering studies in Sweden showed that increased dietary intake of retinol and increased serum levels of retinol were associated with decreased bone mineral density (BMD) and increased susceptibility to fractures (44, 112). Since then, many studies have been performed to investigate whether these studies can be confirmed in other cohorts, but the results are divergent. Some studies have reproduced the initial studies reporting a detrimental effect by vitamin A on bone, but other have found either no association or a beneficial effect (2, 113–115).

Vitamin A status is best assessed by quantifying liver stores, but for obvious reasons such assessments are not useful for clinical studies. In most studies, serum concentrations of retinol are used as a marker for vitamin A status, although retinol levels in serum are tightly regulated and best used only for assessments of deficiency or excess of vitamin A (116, 117). For these reasons, analysis of serum retinol is not recommended for use on an individual basis but is considered useful at the population basis (48). A more relevant marker of vitamin A status is serum levels of retinyl esters, but these analyses are less frequently used (118). Also analyses of ß-carotene in serum have been used in several studies, often in comparison to analyses of retinol. In addition to serum markers, several studies are based on assessments using questionnaires of dietary intake of retinoids, although the intake of vitamin A is difficult to assess since it may be associated with intake of many other nutrients which may not always be easy to adjust for.

## Epidemiological studies indicating a harmful effect by vitamin A on the skeleton

### Studies indicating a negative association between intake of vitamin A and bone mineral density

The first clinical investigation indicating a negative association between intake of vitamin A and BMD was the study by Melhus et al. showing that intake of retinol in 175 women (28-71 yrs) was associated with decreased BMD in femoral neck, lumbar spine and total body (**Table 1**) (44). Three other studies have also observed a negative association between intake of retinol and BMD or decrease of BMD over time in either femoral neck or lumbar spine, although this was not consistently found for all sites examined (**Table 1**) (119, 120, 122). In one study, the association was lost when vitamin A supplements were added (119). In another study was observed that in participants not using vitamin A supplements there was an positive association between dietary vitamin A intake and BMD, and changes of BMD in hip and lumbar spine during the 4-years follow-up, whereas intake of vitamin A was inversely related to BMD and BMD changes in those which used supplemental retinol (120).

Table 1Vitamin A intake and bone mineral density.

**Table:** 

Negative associations			
Individuals	BMD assessments	Associations	Ref.
175 women 28-71 yrs	DXA lumbar spine femoral neck trochanter	Retinol intake negatively associated with BMD femoral neck (*P*=0.05), lumbar spine (*P*=0.001) and total body (*P*=0.009).	(44)
891 women 45-55 yrs	DXA lumbar spine femoral neck with a follow-up after 5-7 yrs	Retinol intake negatively associated with greater femoral neck decrease of BMD (r=-0.067; *P*<0.05) but not with lumbar spine BMD.	(119)
570 women 380 men 55-95 yrs	DXA femoral neck hip lumbar spine with a follow-up after 4 yrs	Low BMD and decrease of BMD associated with both low and and high intake of retinol in both men and women; strongest association for femoral neck and weakest for lumbar spine.	(120)
3,052 women 50-70 yrs	DXA distal forearm	BMD higher in women not given cod liver oil during childhood compared to those forced to take the oil.	(121)
150 postm. women >45 yrs	DXA lumbar spine total body total femur femoral neck	Vitamin A intake negatively associated with BMD lumbar spine (r=-0.201; *P*<0.013) but not with BMD other sites.	(122)

**Table:** 

No associations			
Individuals	BMD assessments	Associations	Ref.
2,016 perim. women 48-52 yrs	DXA femoral neck lumbar spine with a follow-up after 5 yrs	No association between intake of retinol or vitamin A (retinol equivalents) and BMD or change of BMD over time.	(123)
11,068 women 50-70 yrs	DXA total hip spine total body	No association between intake of retinol or total vitamin A and BMD.	(124)
447 women 470 men 69-79 yrs	DXA total hip with a follow-up after 2-5 yrs	No association between vitamin A intake and decrease of BMD over time.	(125)
189 postm. women 50-75 yrs	DXA lumbar spine femoral neck total hip	No association between retinol intake and BMD.	(126)
58 women 45-75 yrs	DXA trochanter	No association between retinol intake and BMD.	(127)
31 osteop. women 29 controls	DXA proximal femur spine	No difference in vitamin A intake between the groups.	(128)

**Table:** 

Positive associations			
Individuals	BMD assessments	Associations	Ref.
3,116 women 2,172 men >55 yrs	DXA femoral neck	Vitamin A and retinol intake positively associated with BMD	(129)
3,574 women 2,907 men >50 yrs	DXA total hip femoral neck lumbar spine	Vitamin A intake positively associated with BMD total hip and femoral neck in men and with lumbar spine in women; not observed in men with low serum 25(OH)D3 (<50 nmol/L)	(130)
2,101 women 1,053 men 40-75 yrs	DXA lumbar spine total hip femoral neck	Vitamin A intake positively associated with BMD total hip and femoral neck	(131)
110 women >65 yrs	DXA T-scores	Vitamin A intake lowest in individuals with T-score ≤ -2.5	(132)
189 postm. women 50-75 yrs	DXA total hip lumbar spine femoral neck	Vitamin A intake positively associated with BMD at all locations	(126)
160 postm. women 50-85 yrs	DXA lumbar spine femoral neck	Vitamin A intake positively associated with BMD lumbar spine but not femoral neck	(133)
66 prem. Women 28-39 yrs	DXA total body lumbar spine femoral neck	Vitamin A intake positively associated with BMD	(134)

In a Norwegian study enrolling 3,052 women, BMD in forearm was found to be higher in women who had not been given cod liver oil during childhood, compared to those which had been forced to consume the oil (**Table 1**) (121).

Interestingly, one study found that not only high intake of retinol, but also low intake of retinol was associated with low BMD and greater change of BMD over time (**Table 1**) (120).

### Studies indicating a negative association between intake of vitamin A and fracture risk

Although low BMD, as assessed by DXA, is a well-established risk factor for osteoporotic fractures, the incidence of low-trauma fractures in spine, hip and forearm are the clinically most relevant symptoms, with hip fractures being the most serious event. In one study enrolling 1,124 women with 247 hip fracturs, high intake of retinol was associated with increased risk for fracture (**Table 2**) (44). In another study enrolling 1,221 men with 111 fractures at any site, those with highest quantile of retinol intake had increased risk for fracture compared to those in the lowest quantile (112). In the Nurse´s Health study enrolling 72,337 postmenopausal women with 603 incident hip fractures, highest quantile of total vitamin A intake or retinol intake was significantly associated with increased fracture rate (135). The risk was further enhanced in those taking vitamin A supplements. Lim et al. have also observed an increased hip fracture risk in those using vitamin A supplements although the intake of vitamin A or retinol was not associated with increased risk of hip fracture (140).

Table 2Vitamin A intake and fracture risk.

**Table:** 

Negative associations			
Individuals	Fractures (fx)	Associations	Ref.
247 women 877 controls 40-76 yrs	247 hip fx	High retinol intake positively associated with hip fx.	(44)
1,221 men	111 all fx	Highest quantile for retinol intake (>1.50 mg/d) had an increased risk of fracture compared to lowest quantile (<0.5 mg/d).	(112)
72,337 postm women 34-77 yrs	603 hip fx	Highest quantile of total vitamin A intake (≥3,000 µg/d retinol equivalents) had significantly increased risk for hip fx (RR 1.48; *P*<0.003) compared to those in lowest quantile (<1,250 µg/d RE). Similar observation made for retinol intake.	(135)

**Table:** 

No associations			
Individuals	Fractures (fx)	Associations	Ref.
34,703 postm. women	521 hip fx 6,502 all fx	No significant associations across quantiles of total or retinol intake with fracture risk.	(136)
2,016 perim. women	140 fx append. skeleton 26 vertebr. fx including 3 ind. with both fx types	Analyses of retinol and total vitamin A intake in quantiles did not show any increased fracture risk.	(123)
75,747 postm. women 50-79 yrs	588 hip fx 10,405 all fx	Analyses of retinol and total vitamin A intake in quantiles did not show any increased fracture risk.	(137)
26,749 women 7,947 men 20-89 yrs	1,555 women and 343 men one or more fx	No significant association between intake of retinol and fracture risk.	(138)

**Table:** 

Positive associations			
Individuals	Fractures (fx)	Associations	Ref.
726 cases mean age 70 yrs comp. to 726 controls mean age 71 yrs	726 hip fx	High retinol intake positively associated with reduced fracture risk	(139)

### Studies indicating a negative association between serum levels of vitamin A and bone mineral density

Two studies have observed that high serum levels of retinol are associated with low BMD in lumbar spine, femoral neck or hip (**Table 3**) (141, 142). In one study, high maternal retinol was associated with low total body mineral content and bone area in offspring but, however, not with BMD (143).

Table 3Serum concentrations of retinol and bone mineral density.

**Table:** 

Negative associations				
Individuals	BMD assessments	Retinol concentrations (µmol/L)	Associations	Ref.
222 postm. women Mean age 57.4 yrs	DXA lumbar spine total hip	1.17 – 5.65 Average 2.7 ± 0.86 >2.4 in 36.4%	T-score LS and hip BMD significantly decreased in Q5 (*P*<0.001) and Q4 (*P*<0.01) *vs* Q1	(141)
154 postm. women >65 yrs T-score < -2.5 femoral neck and/or lumbar spine	DXA lumbar spine femoral neck	Q1; <1.7 Q3; 1.95-2.14 Q5; >2.43	Negative correlation retinol and BMD LS (R=-0.210; *P*<0.01) and FN (R=-0.324; *P*<0.001)	(142)
520 mother-offspring pairs 30.7 ± 3.9 yrs	DXA offspring total body 2 wks after birth	Average 1.31 ± 0.36	High maternal retinol associated with low offspring total body BMC (*P*=0.02) and bone area (*P*=0.009) but not with BMD	(143)

**Table:** 

No associations				
Individuals	BMD assessments	Retinol concentrations (µmol/L)	Associations	Ref.
520 mother-offspring pairs 30.7 ± 3.9 yrs	DXA offspring total body 2 wks after birth	Average 1.31 ± 0.36	No association maternal retinol with offspring total body BMD	(143)
5,790 men and women >20 yrs	DXA femoral neck, trochanter, intertrochanter, total hip	Retinyl esters as % of total serum vitamin A 32.8%≥10%	No significant association retinol assessment and BMD at any site	(144)
59 postm. women 30 w osteop. 29 wo osteop.	DXA lumbar spine, proximal femur	Retinyl esters as % of total serum vitamin A	No significant association retinol assessment and BMD at any site	(128)
78 young men 22.6 + 0.7 yrs	DXA total body, lumbar spine, hip	2.43 ± 0.49	No significant association retinol and BMD at any site	(145)

**Table:** 

Positive associations				
Individuals	BMD assessments	Retinol concentrations (µmol/L)	Associations	Ref.
75 osteop. women 75 controls >60 yrs	DXA proximal femur	Osteop. 2.14 ± 0.22 Contr. 2.37 ± 0.22	Retinol positively correlated to femur neck BMD (r=0.36; *P*<0.01)	(146)
45 osteop. women 45 controls >60 yrs	DXA proximal femur	Osteop. 2.02 ± 0.12 Contr. 2.29 ± 0.14	Retinol positively correlated to femur neck BMD (r=0.40; *P*<0.01)	(147)
2,606 women >75 yrs	DXA proximal femur	Range 1.98-2.06 smokers 1.74-2.00 nonsmok.	Retinol positively but weakly correlated to total hip BMD (r=0.09; *P*<0.002)	(148)
2,101 women 1,053 men 40-75 yrs	DXA lumbar spine femoral neck total hip	Q1 = 1.158 Q2 = 1.469 Q3 = 1.718 Q4 = 2.204	Retinol positively correlated to total hip BMD (Q3 and Q4 *vs* Q1; *P*<0.001)	(131)

### Studies indicating a negative association between serum levels of vitamin A and fracture risk

A Swedish study enrolling 2,322 men and an American study enrolling 2,799 postmenopausal women, expiring 84 and 172 hip fractures, respectively, found that high serum retinol increased the risk of hip fractures (**Table 4**) (112, 149). Interestingly, an increased risk for hip fracture was also observed in one of the studies with low levels of serum retinol (149).

Table 4Serum concentrations of retinol and risk of fractures.

**Table:** 

Negative associations				
Individuals	Fractures (fx)	Retinol concentrations (µmol/L)	Associations HR (95% CI)	Ref.
2,322 men medium age 49 yrs in all quantiles 84 hip fractures	Q1; 17 hip fx Q3; 10 hip fx Q5; 22 hip fx	Q1; <1.95 (n=412) Q3; 2.17-2.36 (n=407) Q5; >2.64 (n=408)	Q1 vs Q3 *(ref. group)*: **Hip fx** HR=1.33 (0.60-2.97) Q5 vs Q3: **Hip fx** HR=2.47 (1.15-5.28)	(112)
2,799 postm. women 50-74 yrs 172 hip fractures	Q1; 39 hip fx Q3; 21 hip fx Q5; 43 hip fx	Q1; <1.62 (n=552) Q3; 1.90-2.13 (n=533) Q5; >2.56 (n=543)	Q1 vs Q3 *(ref. group)*: **Hip fx** HR=1.9 (1.1-3.3) Q5 vs Q3: **Hip fx** HR= 2.1 (1.2-3.6)	(149)

**Table:** 

No associations^§^				
Individuals	Fractures (fx)	Retinol concentrations (µmol/L)	Associations HR (95% CI)	Ref.
2,606 women >75 yrs 92 hip fractures	Q1; 26 hip fx Q2; 34 hip fx Q3; 20 hip fx Q4; 28 hip fx	Q1; <1.58 (n=90) Q2; 1.58-1.94 (n=99) Q3; 1.94-2.34 (n=89) Q4; >2.34 (n=91)	Q2 vs Q1 *(ref. group)*: **Hip fx** HR=1.25 (0.91-1.75) Q3 vs Q1: **Hip fx** HR=0.74 (0.49-1.11) Q4 vs Q1: **Hip fx** HR=1.03 (0.72-1.47)	(148)
335 women 663 men 15-80 yrs Mean men 52 yrs Mean women 48 yrs	54 osteop. fx	T1; 0.3-2.8 (n=)? T2; 2.8-3.4 (n=)? T3; 3.4-19.3 (n=)?	T1 vs T2 *(ref. group)*: **Osteop. fx** HR=1.07 (0.52-2.21) T3 vs T2: **Osteop. fx** HR=1.18 (0.58-2.38)	(150)
21,744 men and women 65-79 yrs 1,154 hip fractures	Q1; 294 hip fx Q3; 210 hip fx Q5; 173 hip fx	Q1; <2.1 (n=284) Q3; 2.56-2.97 (n=281) Q5; >3.63 (n=284)	Q1 vs Q3 *(ref. group)*: **Hip fx** HR=1.41 (1.09-1.82) Q5 vs Q3: **Hip fx** HR=1.02 (0.77-1.34)	(151)

§ The study by Sower et al. (143) not included since an old method to analyse retinol was used giving another range of concentrations.

## Epidemiological studies indicating no harmful effect by vitamin A on the skeleton

### Studies indication no association between intake of vitamin A and bone mineral density

In three studies enrolling 2,016, 11,068 and 917 individuals, respectively, no association between intake of retinol or vitamin A and BMD at several sites (femoral neck, lumbar spine, total hip, total body) or decrease of BMD over time was observed (**Table 1**) (123–125). No association between retinol intake and BMD was also found in three studies with low number of individuals (126–128).

Total hip BMD was not found to be associated with multivitamin supplements in a study of 2,606 women older than 75 years of age (148). Similarly, Sowers et al. did not find any association between radial bone mass and use of vitamin A supplements (152).

In several studies, intake of ß-carotene has been separately assessed. In a metanalysis was concluded that ß-carotene intake was not associated with the overall risk of osteoporosis (most frequently assessed by analysis of BMD, but in some studies by fracture risk). In a subanalysis, however, there was a negative association between ß-carotene intake and osteoporosis in Asian women and men, which was not observed in the Western population (153).

### Studies indicating no association between intake of vitamin A and fracture risk

In three studies enrolling 34,703, 75,747 and 34,696 individuals, respectively, there was no association between intake of retinol or vitamin A and fracture risk in hip or with non-hip fractures (**Table 2**) (136–138). Similar finding was made in a fourth smaller study (123). In one study, there was a 17% increased risk for hip fractures, but not for non-hip fractures, among the 12,293 vitamin A supplement users (136).

A Danish study did not find any increased risk of total fractures or fractures in forearm, hip and spine in patients treated with vitamin A analogues (isotretinoin, acitretin) (154).

### Studies indicating no association between increased serum levels of vitamin A and bone mineral density

In one large study enrolling 5,790 women and men and in a smaller study with 59 postmenopausal women analyzing vitamin A status as retinyl esters as % of total vitamin A (retinol + retinyl esters) in serum, no association between vitamin A and BMD in lumbar spine and proximal femur could be observed (**Table 3**) (128, 144), similar to an old study analyzing serum retinol (152).

No association between maternal serum retinol and total body BMD in offsprings analyzed two weeks after birth (143).

Serum retinol and serum RBP4 have been analyzed in 78 young men 23 years of age and found not to be associated with BMD total body, lumbar spine or hip, indicating no association between serum levels of vitamin A and peak bone mass (145).

### Studies indicating no association between serum levels of vitamin A and fracture risk

In an old study by Sowers et al., no association between serum levels of retinol and osteoporotic fractures was found (152). Three subsequent studies were also unable to find that increased serum levels of retinol are associated with increased risk for hip fracture och osteoporotic fractures in general (**Table 4**) (148, 150, 151). In two of the studies, the number of fractures were modest with 92 hip fractures and 54 osteoporotic fractures (148, 150) but the third study included 1,154 hip fractures (151). In the study by Holvik et al., however, there was a modest increased risk of hip fracture (RR 1.41) in the lowest quantile compared to the middle quantile of serum retinol (151).

## Epidemiological studies investigating the relative roles of vitamin A and vitamin D för bone mineral density and fracture risk

In some studies, the potential interaction between vitamin A and D on bone mass and fracture has been investigated. One reason is that low levels of vitamin D are well recognized to be associated with decreased BMD and enhanced fracture risk. Another reason is that vitamin A interferes with vitamin D-dependent intestinal absorption of calcium (155). Since the receptor for vitamin D (VDR) and the RARs binding the biologically active metabolite of vitamin A (ATRA) dimerize with the same receptors (RXR) for regulating DNA transcription (2), it might be that vitamin A and D may interact to regulate BMD and fracture risk.

Although data from the Iowa Women´s Health Initiative Study enrolling 34,703 post-menopausal women did not find an association between retinol intake and fracture risk, such an association was observed in those with highest intake of retinol and low vitamin D (140). Similar observations were made by in a large cohort of 75,747 post-menopausal women participating in the Women´s Health Initiative Observational Study (137). In a Spanish study of 232 postmenopausal women, those with vitamin D-deficiency (25(OH)-D3 <20 ng/ml) and high serum retinol had substantially lower BMD compared to those with low serum retinol (136). In contrast, Holvik et al. could not observe any significant interaction between serum concentrations of 25(OH)-D3 and serum retinol on hip fractures (n=1,154) in a cohort of 21,774 Norwegian women and men (65-79 yrs) (151).

## Epidemiological studies indicating a beneficial effect of vitamin A on the skeleton

### Studies indicating a positive effect by vitamin A intake and bone mineral density

In contrast to studies showing a harmful or no effect by increased vitamin A on the skeleton, there are several studies reporting a beneficial effect (**Table 1**). In a large prospective cohort, The Rotterdam study with 2,172 men and 3,116 women older than 55 years, a positive association between femoral neck BMD and risk of all fractures in the subjects with highest quintile of total vitamin A or retinol intake was observed (129). Such association was not observed with the intake of ß-carotene. The positive association between vitamin A intake and fracture risk was lost after adjustment for BMD. Interestingly, the association between vitamin A intake and fracture risk was seen only in overweight subjects (BMI>25 kg/m^2^). It was speculated that vitamin A may be stored in fat cells and thereby protect the skeleton from harmful effects by vitamin A, but it was also suggested that subjects with high BMI have larger BMD due to more mechanical loading. No significant associations were observed between dietary intake of vitamin D, or plasma levels of vitamin D, and BMD or fracture risk.

Similar to the findings by de Jonge et al., a positive association between vitamin A intake and BMD of total hip, femoral neck and lumbar spine has been observed in two other studies enrolling 6,481 and 3,154 individuals, respectively(**Table 1**) (130, 131). Also in four other studies with fewer number of individuals, a positive effect on BMD by vitamin A intake has been observed (126, 132–134).

### Studies indicating a positive effect by vitamin A intake and fracture risk

One study has found that high intake of retinol is associated with decreased hip fracture risk (**Table 2**) (139).

### Studies indicating a positive effect by increased serum retinol on bone mineral density

There are two large studies enrolling 2,606 women and 3,154 women and men, respectively, which have found that serum retinol is positively associated with total hip BMD (**Table 3**) (131, 148). Also two smaller study have observed that serum retinol is associated with femoral neck BMD (146, 147).

## Meta-analyses of the association between vitamin A and fracture susceptibility

In one meta-analysis examining the relationship between fracture risk and vitamin A (156), eight prospective studies were included in which data for intake of vitamin A, retinol or ß-carotene were available (44, 123, 135, 137, 138, 140, 157, 158). In two of these studies, only data for intake of ß-carotene was available (157, 158). In the four studies where intake of vitamin A or retinol had been recorded the numbers of hip fractures were 247, 603, 525 and 588 (44, 135, 137, 140). In three studies, the numbers of total fractures were 163, 1040 and 1898 (123, 137, 138). The analyses showed that high intake of vitamin A and retinol increased the risk of hip fracture with an adjusted relative risk of 1.29 and 1.40, respectively, whereas high intake of ß-carotene did not increase the risk of hip fracture. High intake of vitamin A and retinol did not affect the risk of total fracture.

Four prospective studies were identified in which serum retinol levels and fractures were available (112, 148–150). In three studies in which serum retinol and hip fractures were available, the numbers of hip fractures were 84, 92 and 172 (112, 148, 149) and in three studies data for serum retinol and total numbers of fractures (123, 266 and 312) were available (112, 148, 150). When analyzing relationship between serum levels of retinol and hip fracture the results demonstrated a U-shaped relationship with increased relative risk at both low (RR 1.56) and high (RR 1.87) levels of retinol, with no association for total fractures. The authors calculate that serum retinol concentration between 1.99 and 2.31 µmol/l seems to be optimal for bone health.

In a second meta-analyses by Zhang et al. (159), published three years after the study by Wu et al. (156), also eight studies examining the relationship between fracture risk and intake of vitamin A, retinol and ß-carotene were included (44, 123, 129, 135, 137, 138, 140, 158). One of the studies in the meta-analysis by Wu et al. was missing in this study (157), but a study by de Jonge et al. was included (published after the meta-analyses by Wu et al.) (129). In the meta-analyses by Zhang et al., higher intake of vitamin A and retinol was found to be associated with an increase of hip fracture risk (RR 1.29 and 1.40, respectively), similar to the analyses by Wu et al. In contrast, high intake of vitamin A and retinol was associated with a slightly decrease of total fracture risk (RR 0.94 and 0.95, respectively); no such association was observed by Wu et al.

The analysis of a relationship between fracture risk and serum levels of retinol included five studies (112, 148–151) of which four were identical to the studies included by Wu et al. (156). One more study by Holvik et al. was included (151), which was published after the analyses by Wu et al. Like Wu et al., the authors found that low levels of serum retinol slightly increased risk of total fracture (RR 1.11) or hip fracture (RR 1.27), but in contrast to Wu et al., no significant association between high serum retinol and fracture risk was found which may be due to the inclusion of the large study by Holvik et al. with 1,154 fractures which did not find any association between serum high serum retinol and hip fracture risk but a modest increased risk at low serum retinol (151).

Interestingly, subgroup analysis of retinol intake and total fracture risk showed that women have a larger fracture risk compared to a subgroup of men and mixed gender and that Europeans have a lower fracture risk than Americans.

## Comments

It is apparent that there is no consensus regarding the potential role of dietary intake of vitamin A and BMD. One problem with these studies is that there is usually a substantial difference in time between the dietary assessments and the measurements of BMD. Another issue is that intake of vitamin A may be associated with intake of other nutrients which are difficult to adjust for. Some studies indicate that the intake of supplements containing retinol affects the outcome and this is not taken into consideration in all studies. Meta-analyses have found that also low intake of vitamin A is associated with low BMD although this has not been observed in all studies. This brings into question if differences between studies might be influenced by differences in the use of supplements and the degree of fortification of cereals and dairy products in different countries. The absorption and metabolism of retinyl esters and ß-carotene as well as the response in target cells are regulated by several enzymes, binding proteins and receptors which all are modulated by genetic variations in the genes encoding these proteins which all most likely influence an interindividual variability in the bioavailability and response to vitamin A. Although not very much studied, genetic variations in genes encoding RBP4, TTR and BCO1 have been shown to be associated with fasting serum retinol levels (48).

Although preclinical studies have clearly demonstrated the harmful effect on cortical bone mass and strength, it is also apparent that there is no consensus regarding the associations between serum retinol and BMD or hip fracture risk in clinical studies. Two meta-analyses have found that low serum retinol is associated with increased hip fracture susceptibility, but in only one of these analyses an association between hip fracture risk and high serum retinol was observed. It should be pointed out that these meta-analyses are based upon few numbers of publications with rather few numbers of hip fractures included. There is obviously a need for more well-powered investigations, which also would make it possible to perform stratified analyses and to determine possible sex and age specific associations and to analyze if there is a threshold for serum retinol and putative negative associations with fracture susceptibility. Since observed associations are very informative but cannot prove causality, there is also a need to use genetic instruments for the serum levels of retinol and to perform 2-sample Mendelian Randomization studies to determine their causal association with BMD and fracture risk.

The observation demonstrating that the bone anabolic response to mechanical loading in mice is decreased by vitamin A should be confirmed in other studies and extended by studies aiming to elucidate the potential effect by vitamin A on the response in the skeleton to physical activities and to investigate if vitamin A affects forced bone formation induced during bone fracture healing or by treatment of osteoporotic patients with bone anabolic drugs.

Although one study failed to observe an association between serum retinol and BMD in young men (23 yrs of age) (145), the importance for the growing skeleton of an adequate intake of vitamin A is indicated by recent observations in 426 Chinese children (6-9 yrs) showing that serum retinol and BMD are related by an invert U-shaped association. The existence of a reflection point shows the importance of not overdosing the intake of vitamin A (160).

## Author contributions

UL: Conceptualization, Writing – original draft, Writing – review & editing.
